# Conversion of Exhausted Sugar Beet Pulp into Fermentable Sugars from a Biorefinery Approach

**DOI:** 10.3390/foods9101351

**Published:** 2020-09-24

**Authors:** Cristina Marzo, Ana Belén Díaz, Ildefonso Caro, Ana Blandino

**Affiliations:** Department of Chemical Engineering and Food Technology, Faculty of Sciences, IVAGRO, University of Cádiz, Campus Universitario de Puerto Real, 11510 Puerto Real, Spain; cristina.marzo@gm.uca.es (C.M.); ildefonso.caro@uca.es (I.C.); ana.blandino@uca.es (A.B.)

**Keywords:** enzymatic hydrolysis, solid-state fermentation, sugar beet, sugars hydrolysate, generic microbial feedstock

## Abstract

In this study, the production of a hydrolysate rich in fermentable sugars, which could be used as a generic microbial culture medium, was carried out by using exhausted sugar beet pulp pellets (ESBPPs) as raw material. For this purpose, the hydrolysis was performed through the direct addition of the fermented ESBPPs obtained by fungal solid-state fermentation (SSF) as an enzyme source. By directly using this fermented solid, the stages for enzyme extraction and purification were avoided. The effects of temperature, fermented to fresh solid ratio, supplementation of fermented ESBPP with commercial cellulase, and the use of high-solid fed-batch enzymatic hydrolysis were studied to obtain the maximum reducing sugar (RS) concentration and productivity. The highest RS concentration and productivity, 127.3 g·L^−1^ and 24.3 g·L^−1^·h^−1^ respectively, were obtained at 50 °C and with an initial supplementation of 2.17 U of Celluclast^®^ per gram of dried solid in fed-batch mode. This process was carried out with a liquid to solid ratio of 4.3 mL·g^−1^ solid, by adding 15 g of fermented solid and 13.75 g of fresh solid at the beginning of the hydrolysis, and then the same amount of fresh solid 3 times every 2.5 h. By this procedure, ESBPP can be used to produce a generic microbial feedstock, which contains a high concentration of monosaccharides.

## 1. Introduction

The production of high-value-added products, such as organic acids, vitamins, enzymes, bulk chemicals, biofuels, etc. through biotechnological processes using agro-industrial lignocellulosic residues (wheat straw, sugarcane bagasse, sweet sorghum bagasse, etc.) as raw material has become more interesting over the years [1]. They do not compete with food supply, they are generated in big volumes, and they are a sustainable and abundant renewable resource [2,3].

For the conversion of lignocellulose to fermentable sugars, a hydrolysis step is required. This step can be carried out chemically or enzymatically, although the latter shows the added advantage of not generating toxic compounds and being more environmentally friendly [4], while chemical hydrolysis needs several steps to completely remove the chemical from the final products. Moreover, the enzymatic process does not produce unfavorable by-products, the downstream processing is simpler, and corrosion of equipment is avoided [5]. However, the commercial and industrial application of enzymes is limited given their expensive production process and poor stability [6].

Enzyme costs contribute significantly to the economic viability of transforming lignocellulose into bioproducts of interest for the energy industry and the chemical, food, and materials sector. As a result, biomass hydrolysis continues to be a bottleneck of the overall process. Therefore, different strategies, such as the seeking of new sources of enzymes or different hydrolysis technologies have been investigated to make the process more cost effective [7].

One of the most interesting ways for the conversion of cellulose and hemicellulose into reducing sugars for the production of different products is the use of cellulolytic enzyme cocktails of fungal origin [8]. This fact is the consequence of the capability of some fungi to produce hydrolytic enzymes [9]. Therefore, there is a great interest in identifying fungi that secrete these enzymes efficiently by using cheap carbon sources. In this regard, solid-state fermentation (SSF) is an interesting option, because heterogeneous solids, such as agro-food industry wastes and agricultural residues, which are low-value and abundant raw materials, can be used as a solid, support, carbon source and as inducers for fungal growth and enzyme production [10]. Several agri-food residues, such as soybean meal, sunflower, or wheat waste, have been converted into hydrolysates by using crude enzymes produced by the solid-state fermentation of these solid wastes. Other authors have even employed mixtures of residues, such as sugarcane bagasse and soybean hull or food and bakery waste. Afterward, fermented solids were used as a source of enzymes to obtain fermentation feedstocks [11,12,13,14,15].

**Figure 1:**
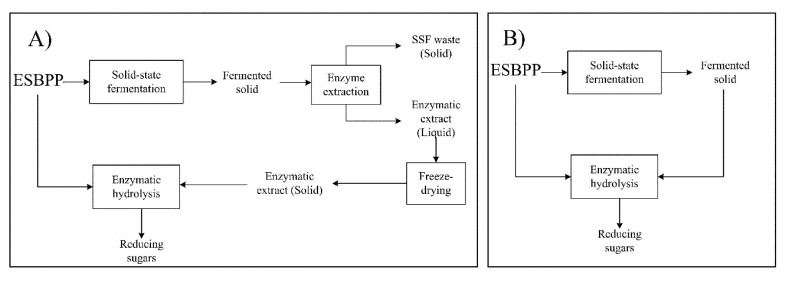
Schemes of the enzymatic hydrolysis of exhausted sugar beet pulp pellets (ESBPPs) by crude enzyme extracts obtained by solid-state fermentation (SSF) (**A**) and by the addition of fermented solid (**B**).

Taking into account the above considerations, the main goal of this study is the production of a hydrolysate rich in fermentable sugars, which could be used as a generic microbial culture medium, using exhausted sugar beet pulp pellets (ESBPPs) as raw material. For this purpose, the hydrolysis was performed through the direct addition of the fermented biomass obtained by SSF as an enzyme source. In this way, the cost of the process could be reduced as enzyme extraction and purification are not required. Figure 1 illustrates the differences between the conventional enzymatic hydrolysis, in which enzymes produced through solid-state fermentation are extracted and lyophilized before their use (Figure 1A) and the one employed in this study (Figure 1B).

ESBPPs are the solid obtained after industrial sugar extraction by diffusion from sugar beets and they are normally dehydrated, granulated, and sold for animal feeding. In this work, firstly, ESBPPs were used as a substrate to produce hydrolytic enzymes using *Aspergillus awamori* solid-state fermentation. This fermented solid, containing the hydrolytic enzyme cocktail secreted by the fungus during its growth, was used for the hydrolysis of fresh ESBPPs. Finally, monosaccharide concentration and carbon to nitrogen (C/N) ratio in hydrolysates were measured to evaluate their potential as a microbial culture medium.

## 2. Materials and Methods

### 2.1. Raw Material

Fresh ESBPPs were obtained from AB Azucarera Iberia (AB Sugar—ABF Group, Jerez de la Frontera, Andalucía, Spain). Samples were collected and stored at 4 °C until use. Detergent fiber analysis of the solid showed that they are mainly composed of pectin (41.13%), cellulose (25.18%), and hemicellulose (16.60%), with the total carbohydrate polymer content being around 83% of the weight [16].

For solid-state fermentation (SSF), fresh ESBPPs were soaked in distilled water (25% w/w) to break up the pellets and dried in an oven at 40 °C for 24 h. For enzymatic hydrolysis experiments, they were directly used in pellet form. Solids were sterilized in an autoclave (120 °C for 20 min) before SSF or enzymatic hydrolysis.

### 2.2. Enzyme Production by Solid-State Fermentation on Fresh ESBPPs

*Aspergillus awamori* 2B.361 U2/1, a sequential mutant of *Aspergillus niger* NRRL 3312, was selected to produce hydrolytic enzymes by solid-state fermentation. Spores were stored in glycerol (50% v/v) at −25 °C. Spores solution was spread on Petri dishes containing a synthetic medium composed by (g·L^−1^): 1 peptone, 0.5 yeast extract, 15 agar, 6 xylan, 5 avicel, and 1 pectin. Petri dishes were incubated at 30 °C for 5 days. After the incubation period, spores were suspended in 0.9% v/v of sodium chloride (NaCl) solution by gentle shaking. The concentration of the spore suspension was calculated using an improved Neubauer counting chamber.

SSF was performed by adding to each disposable Petri dish: 5 g of dried ESBPPs, the volume of spore suspension required to obtain a final inoculum concentration of 1 × 10^7^ spores·g^−1^ of dried solid, and the appropriate amount of a nutrient solution to adjust the initial moisture content to 70% w/w. The nutrient solution composition was as follows (g·L^−1^): 2.4 urea, 9.8 (NH_4_)_2_SO_4_, 5.0 KH_2_PO_4_, 0.001 FeSO_4_·7H_2_O, 0.0008 ZnSO_4_·7H_2_O, 0.004 MgSO_4_·7H_2_O, and 0.001 CuSO_4_·5H_2_O at pH 5.0. pH was not controlled, and Petri dishes were incubated under static conditions at 30 °C for 8 days [16]. For hydrolysis experiments, the whole content of the Petri dishes after eight days of fermentation (fermented ESBPPs) was used as a source of hydrolytic enzymes.

### 2.3. Enzyme Extraction and Activity Assays

The enzymes produced were extracted to determine the enzyme activity. The fermented solid in each Petri dish (5 g) was suspended in 50 mL of 0.1% v/v Tween 80 and incubated for 30 min in a rotary shaker at 4 °C and 150 rpm. Afterward, the resulting solid suspensions were centrifuged at 10,000 rpm and 4 °C for 10 min. The supernatant liquor, the enzymatic extract, was lyophilized and stored until use. Cellulase (FPase, EC 3.2.1.91), xylanase (EC 3.2.1.8), and exo-polygalacturonase (exo-PG, EC 3.2.1.67) activities were measured in the lyophilized enzyme extracts.

An FPase assay was carried out by incubating 0.5 mL of suitable diluted enzyme extract with 1 mL of citrate buffer (0.05 M, pH = 4.8), containing a Whatman No.1 filter paper strip (1 × 6 cm, 50 mg), at 50 °C for 60 min. For xylanase, a reaction mixture containing 0.1 mL of diluted enzymatic extract and 0.9 mL of xylan suspension (0.5% w/v Birchwood xylan in 0.1 M acetate buffer, pH 5.0) was incubated at 50 °C for 10 min. For exo-PG activity, a reaction mixture containing 0.2 mL of diluted enzymatic extract and 0.8 mL of pectin suspension (0.5% w/v in 0.1 M acetate buffer, pH 5) was incubated at 45 °C for 10 min. Reducing sugars (RS) produced after all these reactions were measured by the dinitrosalicylic acid method (DNS) adapted for microplates [17,18]. 

A unit of enzyme activity (U) was defined as the amount of enzyme that produces 1 µmol of reducing sugars per minute under the specified conditions of pH and temperature.

All the assays were made in triplicate.

### 2.4. Enzymatic Hydrolysis of Fresh ESBPPs by Adding Fermented Solid

The enzymatic hydrolysis of fresh ESBPPs was carried out in batch mode in Erlenmeyer flasks (500 mL). Fresh ESBPPs were mixed in different ratios, depending on the experiment, with fermented ESBPPs as a source of hydrolytic enzymes. Both solids were blended into 300 mL of citrate-phosphate buffer (pH 5, 0.05 M). The solid suspensions were continuously mixed by incubating the flasks in an orbital shaker at 200 rpm for 5 days. Samples were withdrawn periodically for RS concentration analysis at different hydrolysis times.

Various studies were performed to enhance the hydrolysis yield. The proportions of fermented and fresh ESBPPs added in each experiment are expressed as a fermented to fresh solid ratio (FFR). This ratio is expressed as grams of initial fermented solid used in SSF per grams of fresh solid added to the hydrolysis (grams of fermented solid:grams of fresh solid).

Firstly, the effect of temperature was assayed at 50 and 55 °C with FFRs of 5:30, 15:15, and 30:0. Secondly, the effect of the fermented to fresh solid ratio was study at 50 °C and at the following FFRs: 5:30, 10:30, 15:30, 15:35, 15:40; 15:45, 15:50, and 15:55. Thirdly, the effect of supplementing the enzymatic hydrolysis with commercial cellulase (Celluclast^®^) (Merck, Darmstadt, Germany) was analyzed in the best condition obtained previously by adding 1.44, 2.17, or 2.89 units of cellulase per gram of dried fresh ESBPPs (U·g^−1^). 

#### High-Solid Hydrolysis in Fed-Batch Mode

High-solid hydrolysis was performed in fed-batch mode to improve the efficiency of the process following the strategies described in Table 1. For strategies I to VI, different amounts of fresh ESBPPs were added to 15 g of fermented ESBPPs at different time intervals. For strategy VII, 5 g of fermented ESBPPs were added in fed-batch mode, at 0, 24, and 48 h, to 55 g of fresh ESBPPs. Celluclast^®^ (2.17 U·g^−1^) and 300 mL of citrate-phosphate buffer were added to all the Erlenmeyer flasks, and they were incubated in a rotary shaker at 200 rpm and 50 °C.

### 2.5. Enzymatic Hydrolysis by SSF Enzymatic Extract

The enzymatic hydrolysis strategy proposed in Section 2.4 was compared to a more conventional methodology, in which the lyophilized enzymatic extract obtained from fungal-fermented ESBPPs was added to fresh ESBPPs. This extract was obtained from the extraction of 15 g of fermented solid (3 Petri dishes).

For this purpose, 55 g of fresh ESBPPs were autoclaved at 120 °C for 20 min in 500 mL Erlenmeyer flasks containing 290 mL of citrate-phosphate buffer 0.05 M at pH 5.0. After that, the lyophilized enzyme extract obtained as described in Section 2.3 was dissolved in 10 mL of citrate-phosphate buffer 0.05 M at pH 5.0 and added to the Erlenmeyer flask. The suspensions were incubated at 50 °C and 200 rpm for 5 days. All the hydrolysis experiments were analyzed in triplicate.

### 2.6. Sample Analyses

RS were measured by the dinitrosalicylic acid method (DNS) adapted to microplate [17,18]. Glucose and arabinose plus galactose concentrations were measured by using enzymatic assay kits from Biosystems (D-Glucose/D-Fructose) (Barcelona, Cataluña, Spain) and Megazyme (L-Arabinose/D-Galactose assay kit) (Bré, County Wicklow, Ireland), respectively. 

Dissolved organic carbon and dissolved nitrogen analyses were carried out in an Analytik-Jena^®^ (Jena, Germany) multi N/C 3100 carbon analyzer with a chemiluminescence detector (CLD) according to the combustion-infrared method (5310B) of the Standard Methods [19].

### 2.7. Parameter Estimation 

The enzymatic hydrolysis of lignocellulosic materials into fermentable sugars is carried out by a complex mechanism that involves diverse phenomena such as adsorption, desorption, enzyme deactivation, etc. In this work, the kinetic model proposed by Caro et al. [20] was used to calculate the general kinetic parameters of the enzyme hydrolysis process: the initial reducing sugar concentration (G_o_); theoretical maximum RS concentration, which is the final reducing sugar concentration to be theoretically reached at the end of the process (G_f_); and the global hydrolysis rate (k_h_). All of them have been calculated for each experiment included here by nonlinear regression, using the damped least squares analysis [21], fitting the experimental data to the equations of the model. The model considers the heterogeneous nature of the process, in which a solid in the form of small particles is decomposed to monosaccharides by the action of three types of enzymes in solution. Because of the extension of the model, we have unified here the three kinetic parameters of the three types of enzymes into only one, resembling a first-order kinetic. Thus, the value of the global rate constant (k_h_) has been obtained as the sum of the three kinetic constants gathered in the model proposed by Caro et al., which corresponds to the activity of the three types of enzymes included in that model. In summary, after obtaining the three mentioned kinetic parameters (G_o_, G_f_, k_h_), we have considered for discussion that the process follows the next simple equation:

(1) $$\frac{G-G_{o}}{G_{f}-G_{o}}=1-e^{-k_{h}t}$$

where G is the concentration of reducing sugars at each instant of time (t) of each experiment.

In order to compare several conditions, a characteristic productivity (P) of the system has been defined as follow: $\left(\mathrm{Gf}\;-\;\mathrm{Go}\right)\cdot k_{h}$. This parameter combines the maximum amount of sugar produced $\left(G_{f\;}\;-{\;G}_{0}\right)$ and the rate of production ($k_{h}$). The regression coefficient (r^2^) of the experimental data to the kinetic model of Caro et al. [20] is also shown.

Yield was calculated by dividing the grams of RS experimentally produced by the theoretical ones.

### 2.8. Statistical Analysis

All experiments and assays were performed in triplicate. Statgraphics 18 was used for data analysis. Data were analyzed using one-way analysis of variance (one-way ANOVA), and Fisher’s least significant differences (LSD, *p* < 0.05) was used to determine significant differences among tested conditions.

## 3. Results and Discussion

### 3.1. Enzymatic Hydrolysis of Fresh ESBPPs by Adding Fermented Solid

The fermented solid obtained after SSF showed a different fiber composition than that of the fresh solid. These results were previously published by Marzo et al. [16]. Briefly, the content of cellulose increased 38% after SSF, whilst hemicellulose and pectin content decreased 33% and 22%, respectively. Furthermore, this fermented solid contained a variety of hydrolytic enzymes secreted by *A. awamori* during its growth. The main enzymatic activities measured in the lyophilized extract obtained from the fermented ESBPPs were as follows: 24.6 ± 0.6 U xylanase·g^−1^ of dried fresh ESBPPs, 9.3 ± 1.1 U exo-PG·g^−1^ of dried fresh ESBPPs, and 1.9 ± 0.2 U FP-ase·g^−1^ of dried fresh ESBPPs. β-glucosidase activity was also assayed, although the activity value was almost nil. This mixture of enzymes has been widely used to hydrolyze lignocellulosic biomass with a high content of hemicellulose, such as, wheat straw, corn stover, or rice straw [22]. Usually, the enzymes produced during the SSF stage are extracted and purified to use them in the hydrolysis stage, in consequence, the price of the produced enzymes is high. To perform a cost-effective process, the fermented solid obtained in SSF was added directly in the hydrolysis stage.

#### 3.1.1. Effect of Temperature

As stated earlier, fermented solid used for hydrolysis experiments contains the fungus and its secreted hydrolytic enzymes. Given that the fungus can consume the monomeric sugars produced in the enzymatic hydrolysis of polysaccharides, its growth must be limited or restricted to avoid the decrease of sugar concentration in the hydrolysate. In this sense, although the most favorable temperature for the growth of the genus *Aspergillus* is around 30 °C and fungal growth generally decreases at temperatures higher than 30 °C, it has been found that some fungus species can grow at temperatures as high as 50 °C [23,24], which is the optimum temperature for cellulase activity. Therefore, given that fungal growth must be restricted and enzymatic hydrolysis must be favored, 50 and 55 °C were the temperatures selected for hydrolysis in this study.

**Figure 2:**
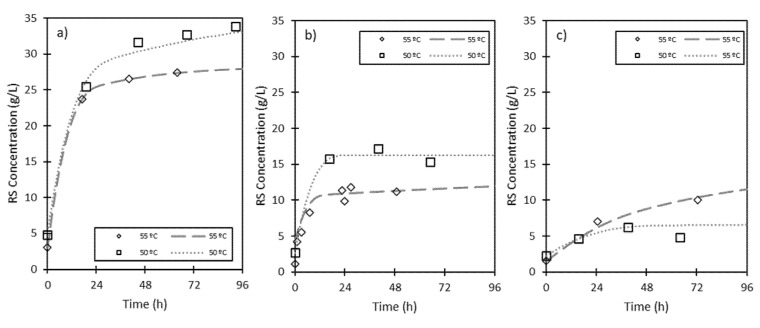
Evolution of reducing sugar (RS) concentration through hydrolysis time fresh ESBPPs in 300 mL of citrate-phosphate buffer (pH 5, 0.05 M). Effect of temperature (T) with various fermented to fresh solid ratios (FFRs): (**a**) 5:30, (**b**) 15:15, and (**c**) 30:0.

Three different fermented to fresh solid ratios (FFRs of 5:30; 15:15, and 30:0) were tested at 50 and 55 °C. The evolution of RS concentration can be observed in Figure 2. When the enzymatic hydrolysis was performed at 55 °C, the hydrolysis rate and the productivity increased compared to those of hydrolysis at 50 °C (see Table 2). However, the theoretical maximum RS concentration (G_f_) and yield decreased when the hydrolysis was performed with FFRs of 5:30 and 15:15 at 55 °C. This reduction was not noticed for an FFR of 30:0 at 55 °C. 

These results reflect the combined effect of sugar production, as a consequence of enzyme hydrolysis, and sugar consumption by the fungus. The amount of fungus is lower in the experiments with FFRs of 5:30 and 15:15, favoring the sugar production, compared with the experiment with an FFR of 30:0, given that a higher quantity of fermented solid is added in the last case. Therefore, it was observed that for FFRs of 5:30 and 15:15, lower RS concentrations were obtained at 55 °C. This behavior can be justified considering the reduction of enzyme activities produced at 55 °C. According to Botella et al. [25], xylanase and exo-PG enzymes produced by *A. awamori* showed an optimum temperature of between 30 and 50 °C, decreasing their activities at higher temperatures and being practically deactivated at 70 °C [25,26]. Other authors have published similar results [8,27]. However, for an FFR of 30:0, the activities of the enzymes are also reduced at 55 °C, but at this temperature, the fungus is also affected and, therefore, the sugar consumption is diminished in these conditions. In this case, the RS concentration obtained is near double at 55 °C than that at 50 °C, which means that a higher temperature hinders the fungus’ growth.

In conclusion, a higher temperature (55 °C) affects fungal growth and, therefore, sugar consumption is reduced, reaching a higher concentration when the FFR is 30:0. However, under an FFR of 15:15, it is recommended to work at 50 °C, given that at this temperature the enzymes are more active.

#### 3.1.2. Effect of Fermented to Fresh Solid Ratio

**Figure 3:**
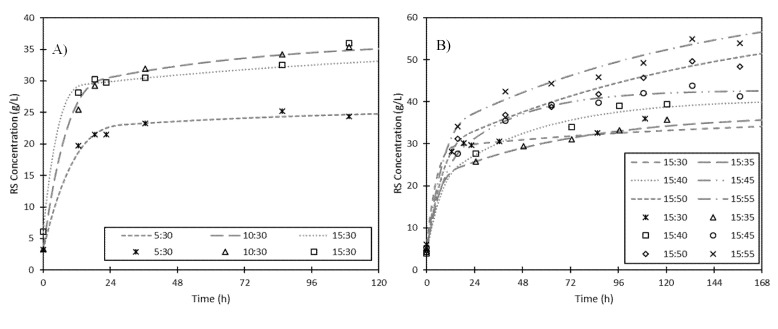
Evolution of RS concentration through hydrolysis time of fresh ESBPPs in 300 mL of citrate-phosphate buffer (pH 5, 0.05 M) at 55 °C with various fermented to fresh solid ratios. FFRs of (**A**) 5:30 (star), 10:30 (triangle), and 15:30 (square) and (**B**) 15:30 (star), 15:35 (triangle), 15:40 (square), 15:45 (circle), 15:50 (diamond), and 15:55 (cross) are shown. Lines indicate theoretical values obtained from the kinetic model and symbols represent experimental values.

To increase the concentration of RS released in the hydrolysis, different fermented to fresh solid ratios (FFRs) were studied, as mentioned in Section 2.4 Two types of experiments were tested: in the first set, different amounts of fermented solid were added to a fixed concentration of fresh solid, while in the second set a fixed amount of fermented solid was added to different amounts of fresh solid. Results are shown in Figure 3 and Table 3.

For the first set of experiments, the FFRs assayed were 5:30, 10:30, and 15:30. As seen in Figure 3A, RS concentration increases rapidly along the first 18 h in all the cases, while afterward the hydrolysis rate is very low. This is a consequence of the two stages (heterogeneous and homogeneous) that form the kinetics involved in the hydrolysis process [20]. The maximum RS concentration increased from 28.4 to 36.5 g·L^−1^ and the hydrolysis yield increased from 27.9% to 32.4% as the fermented solid mass rose from 5 to 10 g. Nevertheless, a similar maximum RS concentration was obtained when the fermented solid mass was 15 g (36.7 g·L^−1^) while the hydrolysis yield decreased until 29.7%. Probably, the addition of more fermented solid does not affect the maximum RS concentration as the amount of potential hydrolysable polysaccharides in the total solid mix remains almost constant. However, the global hydrolysis rate (k_h_) increased as the fermented solid mass did, becoming 0.136, 0.141, and 0.200 h^−1^ for FFRs of 5:30, 10:30, and 15:30, respectively (Table 3). Therefore, although maximum RS concentration did not increase using 15 g of fermented solid, the hydrolysis rate was higher due to the increment in enzyme concentration in the total solid mixture. The same effect was observed by Tsakona et al., who reported that the amount of glucose obtained in flour-rich waste hydrolysate (a confectionery and wheat milling plants by-product) increased when higher enzyme activity was added as fermented solid [12].

For the second set of experiments, the FFRs assayed were 15:30, 15:35, 15:40; 15:45, 15:50, and 15:55. The evolution of RS concentrations is shown in Figure 3B and initial RS concentration, theoretical maximum RS concentration, productivity, hydrolysis rate, and hydrolysis yield can be found in Table 3. In these experiments, the amount of fresh solid increases, so the content of potentially hydrolysable polysaccharides also does, yielding higher RS concentrations. This result suggests that, at the very least, there is enough enzyme activity in the fermented solid to hydrolyze a quantity of fresh solid as high as 3.7 times greater. The highest value was obtained for an FFR of 15:55 (66.0 g·L^−1^) with a hydrolysis yield and productivity of 33.1% and 8.1 g·L^−1^·h^−1^, respectively. Comparing this result with the one obtained for an FFR of 15:30, an increase of 80% in the maximum RS concentration and a reduction in the hydrolysis rate from 0.200 to 0.133 h^−1^ were observed. However, a similar yield was obtained. This behavior is probably related to the decrease in the liquid to solid ratio from 6.7 to 4.3 mL·g^−1^ solid. No greater amounts of fresh solid were tested because the resulting suspension was very difficult to agitate.

#### 3.1.3. Supplementation of Fermented ESBPPs with Commercial Cellulase

**Figure 4:**
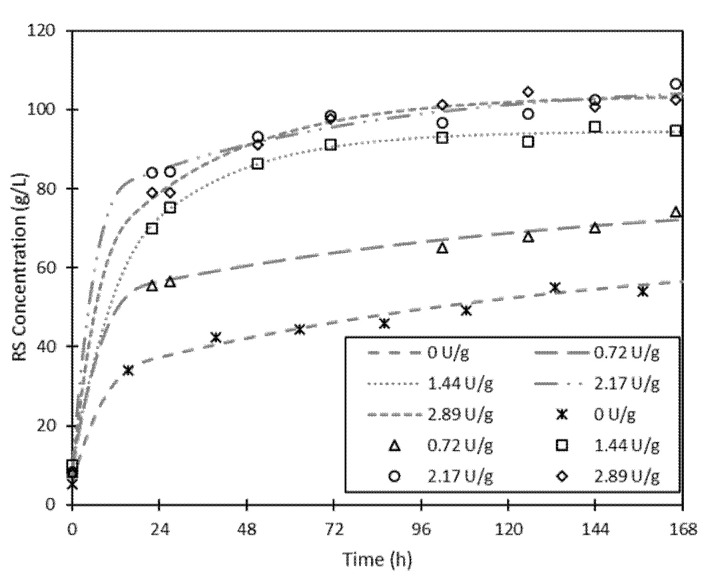
Evolution of RS concentration over hydrolysis time of fresh ESBPPs in 300 mL of citrate-phosphate buffer (pH 5, 0.05 M) at 50 °C with an FFR of 15:55 and with addition of Celluclast^®^ (0, 0.72, 1.44, 2.17, and 2.89 U·g^−1^ of dried fresh ESBPPs). Lines indicate theoretical values obtained from the kinetic model and symbols represent experimental values.

Celluclast^®^, a commercial enzymatic cocktail composed mainly by cellulase, was added as a supplement to the enzymatic hydrolysis to improve hydrolysis yield. The enzyme activities of Celluclast^®^ added to the medium were 0.72, 1.44, 2.17, and 2.89 U·g^−1^ of dried fresh ESBPPs. The reducing sugar concentration obtained is shown in Figure 4, and the theoretical maximum RS concentration, productivity, and hydrolysis rate are presented in Table 4. As the enzymatic activity of Celluclast^®^ increased, the concentration of reducing sugars, the hydrolysis yield and the hydrolysis rate also did. The main reason for this behavior is the low content in cellulase of the enzyme cocktail produced by SSF of fresh ESBPPs. Although fresh ESBPPs are mainly composed of pectin (41.13%), they also contain a high percentage of cellulose (25.18%) and hemicellulose (16.60%) [16]. However, the enzyme cocktail produced by SSF of the fresh ESBPPs is mostly composed of hemicellulases (xylanases) and pectinases. Given the lack of cellulases in the cocktail, it is not possible to completely hydrolyze fresh ESBPPs. As can be seen in Figure 4, when the enzyme cocktail obtained from SSF is supplemented with cellulases, higher RS concentrations were attained. The same conclusion can be made focusing on productivity, hydrolysis yield, or hydrolysis rate. These parameters are higher as cellulase activity increases.

Similar effects were previously observed in two studies carried out by our research group [16,28]. In the first study, ESBPPs were hydrolyzed with Celluclast^®^ or with this cocktail supplemented with commercial xylanase and pectinase [28]. In the second study, a lyophilized enzymatic extract obtained from SSF of orange peels, mainly composed of xylanase and pectinase, was supplemented with Celluclast^®^ and used to hydrolyze orange peels or ESBPPs [16]. In all cases, reducing sugar concentrations increased as Celluclast^®^ was added for the hydrolysis.

Considering a confidence level of 98%, RS values obtained with a cellulase activity of 2.17 and 2.89 U·g^−1^ of dried fresh ESBPPs cannot be considered statistically different. Therefore, it is not possible to increase the maximum concentration of reducing sugars in the hydrolysate, using enzyme doses higher than 2.17 U·g^−1^ of dried fresh ESBPPs. 

#### 3.1.4. High-Solid Fed-Batch Enzymatic Hydrolysis 

From the above-mentioned experiments, a maximum RS concentration of 106.8 g·L^−1^ was reached at 50 °C, with an FFR of 15:55, the addition of 2.17 U of Celluclast^®^·g^−1^ of dried fresh ESBPPs and 300 mL of citrate-phosphate buffer (liquid to solid ratio of 4.3 mL·g^−1^). However, in the literature, some authors suggest that with a ratio lower than 6.6 mL·g^−1^, the hydrolysis yield can decrease due to the high concentration of solids [29,30,31]. When the concentration of solids is high, the mass transfer is hampered and the viscosity increases [32]. The combination of both effects can produce a decrease in the water availability, possible enzyme inhibition by product, and irreversible binding of the absorbed enzyme to the substrate [32].

**Figure 5:**
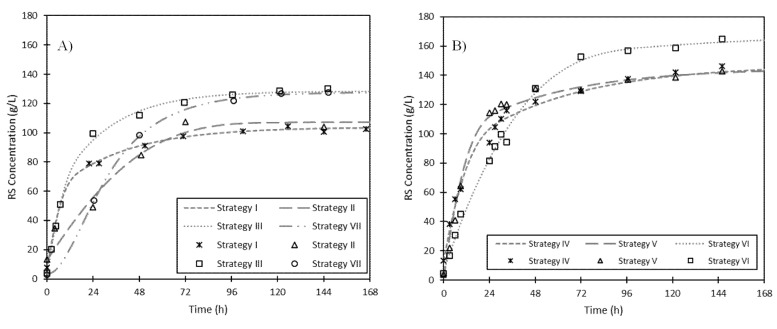
Evolution of RS concentration over hydrolysis time of fresh ESBPPs in 300 mL of citrate-phosphate buffer (pH 5, 0.05 M) at 50 °C with addition of Celluclast^®^ (2.17 U·g^−1^ dried fresh ESBPPs). (**A**) Fed-batch hydrolysis with a total FFR of 15:55. (**B**) Fed-batch hydrolysis with a total FFR of 15:90. Lines indicate theoretical values obtained from the kinetic model and symbols represent experimental values.

Taking into account these considerations, the total mass of fresh solid (55 g) was divided into several fractions, which were added at different times during the hydrolysis (see Table 1). Figure 5A shows the evolution of RS concentrations in the strategies I, II, and III. It can be observed that the highest RS concentration (127.3 g·L^−1^) was obtained with the “strategy III”, in which the fresh solid is added in four fractions. Hydrolysis yield, productivity, and hydrolysis rate were 63.8%, 24.3 g·L^−1^·h^−1^, and 0.2 h^−1^, respectively. Therefore, there was a 22% increase in hydrolysis yield and a 30% increase in productivity compared to that in strategy I, in which the total fresh ESBPPs were added at the beginning of the enzymatic hydrolysis (see Table 5). The viscosity of the hydrolysate decreased significantly when the fresh solid was added in small fractions (“strategy III”), and, as a consequence, agitation and hydrolysis rate improved. Jung et al. also found that high glucose concentrations and saccharification yields could be reached in the enzymatic hydrolysis of maleic acid-pretreated rice straw by the optimization of the fed-batch process, even using high-solid loading [33].

The second set of experiments were performed with a total FFR of 15:90, but the liquid to solid ratio was reduced to 2.9 mL·g^−1^ of solid (Figure 5B). Two different fed-batch strategies were tested (V and VI) and compared to batch mode (IV). Comparing “strategy IV” with “strategy V”, a slight decrease was observed in G_f_, however higher productivity and hydrolysis rates were obtained following the “strategy V” (see Table 5). If these strategies are compared with "strategy VI", in which fresh ESBPPs are added in eight fractions, a G_f_ value of 168.0 g·L^−1^ was achieved and the hydrolysis yield was increased until 54.9%, although productivity and hydrolysis rate decreased (Table 5).

A common problem of enzymatic hydrolysis is the deactivation of the enzymes over time. For this reason, a new strategy (“strategy VII”) was planned, in which the fermented ESBPPs were added every 24 h (FFR of 15:55). Figure 5A shows the RS concentration obtained through “strategy VII” compared with that for “strategy I”, in which all fermented solid was added at the beginning of the hydrolysis. During the first 24 h, “strategy I” produced a higher RS concentration and therefore the hydrolysis rate was higher than the corresponding one of “strategy VII”. However, G_f_ for “strategy VII” was 1.22 times higher than for “strategy I”. Therefore, although the total amount of enzyme added in both experiments was the same, the addition of fermented solid every 24 h improved by 22% both the maximum RS concentration obtained and the hydrolysis yield (see Table 5). 

### 3.2. Evaluation of the Proposed Methodology for ESBPP Hydrolysis

Two experiments were performed by following the processes showed in Figure 1 (process A and B). In the A process, lyophilized crude enzymatic extract was obtained from 15 g of fermented ESBPPs and then it was added to 55 g of fresh ESBPPs in 300 mL of citrate-phosphate buffer (pH 5, 0.05 M) The mixture was incubated at 55 °C and 200 rpm. For the B process, 15 g of fermented solid was directly added to 55 g of fresh ESBPPs as a source of hydrolytic enzymes, maintaining the rest of the conditions as in the A process. Using the A process, G_f_ was 37.8 g·L^−1^, whereas for the B process it was 66.0 g·L^−1^. Moreover, k_h_ increased from 0.105 to 0.133 g·L^−1^·h^−1^ and hydrolysis yield improved from 22.6% to 33.1%. Those differences can be explained considering that the enzyme load in the new proposed strategy is higher than that in the previous strategy. Enzyme losses and deactivation phenomena that normally occur in the concentration and purification stages to obtain enzymatic extracts are avoided by adding fermented solid as a source of enzymes. Moreover, the number of stages required to produce the hydrolysate decreases considerably by using B process. As a result, energy demand, time employed, the chemicals used, and, consequently, the cost of the process can be significantly reduced.

**Figure 6:**
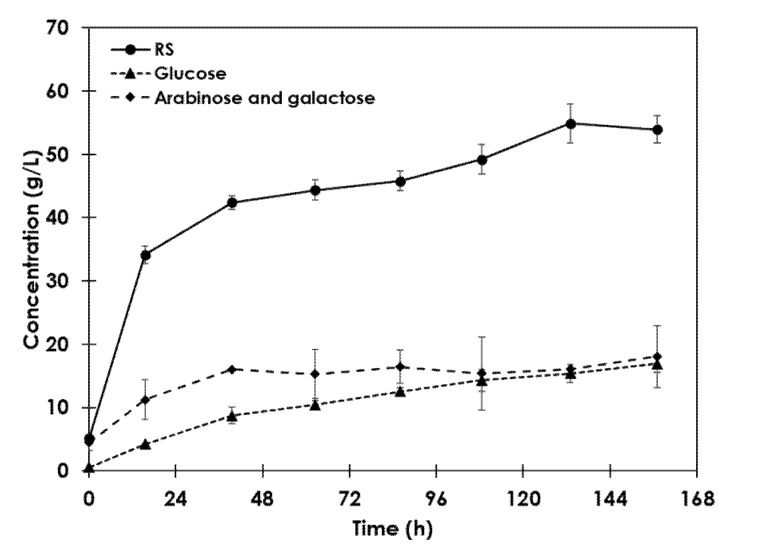
Evolution of reducing sugars concentration (circle), glucose (triangle), and the mixture arabinose and galactose (diamond) in enzymatic hydrolysate.

The hydrolysate obtained by the B process was characterized to know the possible final products that might be obtained through successive sugar fermentation. The temporal evolution of several sugar concentrations in this hydrolysate can be observed in Figure 6. The maximum values measured were as follows: 16.9 g·L^−1^ of glucose and 18.1 g·L^−1^ of a mixture of arabinose and galactose, while the total reducing sugar concentration was 66.0 g·L^−1^.

Most sugars contained in the hydrolysate (glucose and arabinose) are the main sugars consumed by microorganisms to produce value-added products, such as ethanol, lactic acid, etc. In previous studies of our research group, ethanol and lactic acid were produced sequentially from ESBPP hydrolysate, obtained by conventional enzymatic hydrolysis by adding a cocktail of commercial enzymes composed of Celluclast^®^, β-glucosidase, xylanase, and pectinase [28].

In addition to the sugar composition, another important factor in biological processes is the carbon to nitrogen ratio (C/N) [34]. In this case, the C/N measured in ESBPP hydrolysate was 28.80, which is a value that is in the right range for many microbial cultures. For example, bamboo hydrolysate was used by *Clostridium beijerinckii* (ATCC 55025-E604) for ABE (acetone, butanol, and ethanol) production [35]. In this work, the effect of the C/N ratio on butanol production was studied, selecting ratios in the range from 20.89 to 31.98. Results obtained showed that lower or higher C/N ratios result in lower butanol and ABE production because the C/N ratio influences the growth of *Clostridium,* which is responsible for butanol production, with the optimum C/N ratio being 27.5 [35]. It has also been shown that C/N ratio plays an important role in the process of microbial lipid accumulation, and it is generally considered that a C/N ratio higher than 20 is required for the process, with the optimal value varying greatly depending on the cultured microorganism [36]. Similarly, an adequate C/N ratio is fundamental in bio-hydrogen production. Thus, it has been found that in the bio-hydrogen production from raw rice straw using sewage sludge as inoculum, the maximal hydrogen yield was produced at the C/N ratio of 25, which is a value very close to the one of ESBPP hydrolysate [37].

## 4. Conclusions

In the production of value-added products from lignocellulosic biomass, the cost of the enzymes used for the hydrolysis is the bottleneck of the process. To reduce the process cost, enzymes can be produced by SSF using as substrate a sub-product of the agroindustry as raw material (ESBPPs) and this fermented solid can be added directly to fresh ESBPPs for its hydrolysis. In this way, enzyme extraction and purification stages during the enzyme production process are avoided. If enzymatic hydrolysis is conducted at high-solid loading, following a fed-batch strategy, in which fresh solid is added in four fractions, the hydrolysis yield increases by 22% compared to that for the batch strategy, in which the total fresh ESBPPs are added at the beginning of the hydrolysis process. Moreover, analysis of the hydrolysates obtained suggests that a generic microbial feedstock composed mainly by glucose and arabinose can be produced in a more cost- and time-effective process, which can be used to obtain value-added products, such as, biofuels, organic acids, etc.

## Figures and Tables

**Table 1:** Addition strategies used in enzymatic hydrolysis with fermented exhausted sugar beet pulp pellets (ESBPPs).

Strategy Code	Total FFR	Addition Strategy			
		Fresh ESBPPs		Fermented ESBPPs	
I	15:55	55 g	t = 0 h	15 g	t = 0 h
II	15:55	30 g; 25 g	t = 0; 24 h	15 g	t = 0 h
III	15:55	13.75 g (×4)	t = 0; 2.5; 5; 7.5 h	15 g	t = 0 h
IV	15:90	90 g	t = 0 h	15 g	t = 0 h
V	15:90	22.5 g (×4)	t = 0; 3; 6; 9 h	15 g	t = 0 h
VI	15:90	11.25 g (×8)	t = 0; 3; 6; 9; 24; 27; 30; 33 h	15 g	t = 0 h
VII	15:55	55 g	t = 0 h	5 g (×3)	t = 0; 24; 48 h

FFR: fermented to fresh solid ratio.

**Table 2:** Effect of temperature (T) with various FFRs (5:30, 15:15, and 30:0). G_o_: initial reducing sugar (RS) concentration, G_f_: theoretical maximum RS concentration, k_h_: hydrolysis rate (h^−1^), and P: productivity (g·L^−1^·h^−1^).

FFR	T (°C)	G_0_ (g·L^−1^)	G_f_ (g·L^−1^)	k_h_ (h^−1^)	P (g·L^−1^·h^−1^)	r^2^	Yield (%)
5:30	50	5.3	36.8	0.100	3.1	0.991	36.1
5:30	55	3.1	28.4	0.136	3.5	0.999	27.9
15:15	50	2.7	17.0	0.125	1.8	0.987	21.8
15:15	55	1.9	15.4	0.169	2.2	0.967	19.8
30:0	50	2.2	8.2	0.051	0.3	0.999	12.7
30:0	55	1.5	14.5	0.072	0.9	0.993	22.5

**Table 3:** Effect of fermented to fresh solid ratio on hydrolysis experiments performed in 300 mL of citrate-phosphate buffer (pH 5, 0.05 M) at 55 °C with various fermented to fresh solid ratios. G_o_: initial RS concentration, G_f_: theoretical maximum RS concentration, k_h_: hydrolysis rate (h^−1^), and P: productivity (g·L^−1^·× h^−1^).

FFR	G_0_ (g·L^−1^)	G_f_ (g·L^−1^)	k_h_ (h^−1^)	P (g·L^−1^·h^−1^)	r^2^	Yield (%)
5:30	3.1	28.4	0.136	3.5	0.999	27.9
10:30	3.3	36.5	0.141	4.7	0.997	32.4
15:30	6.1	36.7	0.200	6.1	0.997	29.7
15:35	4.3	37.3	0.195	6.4	0.999	26.9
15:40	4.1	40.7	0.179	6.5	0.994	26.5
15:45	5.3	42.9	0.170	6.4	0.995	25.4
15:50	4.7	61.9	0.137	7.8	0.992	33.6
15:55	5.1	66.0	0.133	8.1	0.987	33.1

**Table 4:** Effect of Celluclast^®^ supplementation (0, 0.72, 1.44, 2.17, and 2.89 U·g^−1^ of dried fresh ESBPPs) on hydrolysis experiments performed at 50 °C with an FFR of 15:55. G_o_: initial RS concentration, G_f_: theoretical maximum RS concentration, k_h_: hydrolysis rate (h^−1^), and P: productivity (g·L^−1^·h^−1^).

U/g	G_0_ (g·L^−1^)	G_f_ (g·L^−1^)	k_h_ (h^−1^)	P (g·L^−1^·h^−1^)	r^2^	Yield (%)
0	5.1	66.0	0.133	8.1	0.987	33.1
0.72	8.2	80.0	0.136	9.8	0.996	40.1
1.44	10.1	94.7	0.146	12.4	0.999	47.5
2.17	8.2	106.8	0.183	18.1	0.995	53.6
2.89	6.6	104.0	0.193	18.8	0.997	52.1

**Table 5:** Fed-batch enzymatic hydrolysis strategies (I, II, III, IV, V, VI, VII) at 50 °C with addition of Celluclast^®^ (2.17 U·g^−1^ of dried fresh ESBPP). G_o_: initial RS concentration, G_f_: theoretical maximum RS concentration, k_h_: hydrolysis rate (h^−1^), and P: productivity (g·L^−1^·h^−1^).

Strategy	G_0_ (g·L^−1^)	G_f_ (g·L^−1^)	k_h_ (h^−1^)	P (g·L^−1^·h^−1^)	r^2^	Yield (%)
I	6.6	104.0	0.193	18.8	0.997	52.1
II	13.4	107.7	0.087	8.2	0.962	54.0
III	2.4	127.3	0.195	24.3	0.998	63.8
IV	13.3	147.1	0.113	15.1	0.991	48.1
V	1.3	143.6	0.119	16.9	0.991	47.0
VI	6.8	168.0	0.040	6.4	0.996	54.9
VII	2.9	126.7	0.112	13.9	0.999	63.5
